# Part II: On the Use, the Misuse, and the Very Limited Usefulness of Cronbach’s Alpha: Discussing Lower Bounds and Correlated Errors

**DOI:** 10.1007/s11336-021-09789-8

**Published:** 2021-08-13

**Authors:** Klaas Sijtsma, Julius M. Pfadt

**Affiliations:** 1 Department of Methodology and Statistics TSB, Tilburg University, PO Box 90153, 5000 LE Tilburg, The Netherlands; 2 Ulm University, Ulm, Germany

**Keywords:** classical test theory, coefficient $\alpha$, correlated errors, Cronbach’s $\alpha$, discrepancy of parameters, estimation bias of coefficient $\alpha$, factor-analysis approach to reliability, reliability lower bounds

## Abstract

Prior to discussing and challenging two criticisms on coefficient $\alpha$, the well-known lower bound to test-score reliability, we discuss classical test theory and the theory of coefficient $\alpha$. The first criticism expressed in the psychometrics literature is that coefficient $\alpha$ is only useful when the model of essential $\tau$-equivalence is consistent with the item-score data. Because this model is highly restrictive, coefficient $\alpha$ is smaller than test-score reliability and one should not use it. We argue that lower bounds are useful when they assess product quality features, such as a test-score’s reliability. The second criticism expressed is that coefficient $\alpha$ incorrectly ignores correlated errors. If correlated errors would enter the computation of coefficient $\alpha$, theoretical values of coefficient $\alpha$ could be greater than the test-score reliability. Because quality measures that are systematically too high are undesirable, critics dismiss coefficient $\alpha$. We argue that introducing correlated errors is inconsistent with the derivation of the lower bound theorem and that the properties of coefficient $\alpha$ remain intact when data contain correlated errors.

In a much-cited discussion paper in *Psychometrika*, Sijtsma (2009; 2,415 citations in Google Scholar on 17 May 2021) argued that two misunderstandings exist with respect to coefficient $\alpha$ (e.g., Cronbach, 1951; 51,327 citations, from the same source). First, contrary to common belief, coefficient $\alpha$ is not an index of the internal consistency in the sense of a substantively coherent measure of the same ability or trait. Rather, coefficient $\alpha$ approximates reliability of a score irrespective of the score’s composition. Second, it is little known that coefficient $\alpha$ is a lower bound to the reliability, and that greater lower bounds exist that may be preferable. Based on these observations, Sijtsma (2009) diverted the overwhelming attention for coefficient $\alpha$ to alternative approaches approximating test-score reliability. His take-away message was:

Use $\alpha$ as a lower bound for test-score reliability or use greater lower bounds, but do not use $\alpha$ for anything else.

This message leaves a role for coefficient $\alpha$, but it has not stopped other authors from pouring criticism over coefficient $\alpha$ up to a degree that does not do justice to its usefulness, even if that usefulness is limited (e.g., McNeish, 2018, and Revelle & Condon, 2019, provide overviews; also, Sheng & Sheng, 2012). Given what we consider an unjustified flow of criticism, we think there is room for an article that separates further misunderstandings about coefficient $\alpha$ from what it really is.

In this article, we dissect and reject two frequently presented criticisms on coefficient $\alpha$ that Sijtsma (2009) did not discuss and argue that the criticisms are incorrect. First, we reject the claim that coefficient $\alpha$ is only useful if the items in the test satisfy the mathematical model of essential $\tau$-equivalence (Lord & Novick, 1968, discussed later; e.g., Cho, 2016; Cho & Kim, 2015; Dunn, Baguley, & Brunsden, 2014; Graham, 2006; Teo & Fan, 2013). We argue that models are idealizations of the truth and *by definition* never fit the data perfectly. Hence, the claim that a misfitting model of essential $\tau$-equivalence invalidates the use of coefficient $\alpha$ is reasonable only when one is prepared to reject all results that models imply, a conclusion we expect researchers will rarely entertain. Instead, we will argue that under certain reasonable conditions, coefficient $\alpha$ is a useful lower bound to the reliability irrespective of the fit of the model of essential $\tau$-equivalence. Second, we discuss the claim that theoretically, coefficient $\alpha$ can be greater than the reliability (e.g., Cho & Kim, 2015; Dunn et al., 2014; Green & Hershberger, 2000; Green & Yang, 2009; Lucke, 2005; Teo & Fan, 2013) and argue that this claim is incorrect. To freshen up memory, before we discuss these criticisms and draw conclusions, we start with some theory for coefficient $\alpha$.

The outline of this article is as follows. First we discuss the basics of classical test theory (CTT), including relevant definitions and assumptions, reliability, coefficient $\alpha$, and the theorem that states that alpha is a lower bound to the reliability. Next, we discuss the discrepancy of coefficient $\alpha$ relative to CTT test-score reliability including a discussion of discrepancy from the factor-analysis (FA) perspective, and an examination of the bias in sample estimate ${{\hat{\alpha }}}$ with respect to both parameter $\alpha$ and the test-score reliability. Second, we critically discuss the claims regularly found in the literature that coefficient $\alpha$ is only useful if the items in the test satisfy essential $\tau$-equivalence and that theoretically, coefficient $\alpha$ can be greater than the reliability. We argue that both claims are incorrect. Finally, we summarize the valid knowledge about coefficient $\alpha$.

## Theory of Coefficient ${\alpha }$

Until the 1950s, the dominant method for determining test-score reliability was the split-half method. This method entailed splitting the test into two halves, computing the correlation between the total scores on the test halves as an approximation for the reliability of a test half, and then choosing a correction method for estimating the reliability of the whole test. This method was problematic for two reasons. First, one could split a test in two halves in numerous ways, and even though some rules of thumb existed for how to do this, an undisputed optimal solution was unavailable. Second, given two test halves, several correction methods were available for determining the whole test’s reliability, but agreement about which method was optimal was absent. Amidst this insecurity, Cronbach (1951) argued persuasively that an already existing method (e.g., Guttman, 1945; Hoyt, 1941; Kuder & Richardson, 1937) he renamed coefficient $\alpha$ could replace the split-half method and solve both problems of the split-half method in one stroke. Without reiterating his arguments, Cronbach’s suggestion that coefficient $\alpha$ solves all problems is a perfect example of a message that arrives at the right time when people are most perceptive (but see Cortina 1993; Green, Lissitz, & Mulaik, 1977; Schmitt, 1996, for early critical accounts). Coefficient $\alpha$ became one of the centerpieces of psychological reporting, and until the present day tens of thousands of articles in psychological science and other research areas report coefficient $\alpha$ for the scales they use.

### Coefficient ${\alpha }$ is a Lower Bound to Reliability ${\rho }_{{{X}}{{X}}^{{'}}}$

Because the lower bound result for coefficient $\alpha$ is old and mathematically correct (Novick & Lewis, 1967; Ten Berge & Sočan, 2004), we will not repeat the details here. The CTT model as Lord and Novick (1968; also, see Novick, 1966) discussed it underlies the lower bound theorem; if one does not accept this theory, one may not accept the lower bound theorem. CTT assumes that any observable measurement value $X_{i}$ for subject *i* can be split in two additive parts, a true score $T_{i}$ defined as the expectation of $X_{i}$ across hypothetical independent repetitions, indexed *r* of the measurement procedure, so that

1 $$\begin{aligned} T_{i}=\mathcal {E}_{r}(X_{ir}), \end{aligned}$$

and a random measurement error defined as (e.g., Traub, 1997)

2 $$\begin{aligned} E_{i}=X_{i}-T_{i}, \end{aligned}$$

so that the CTT model is

3 $$\begin{aligned} X_{i}=T_{i}+E_{i}. \end{aligned}$$

Equation (1) provides an operational or syntactic definition of $T_{i}$ (Lord & Novick, 1968, pp. 30–31), liberating it from definitional problems that existed previously in CTT, for example, considering the true score as a Platonic entity typical of the individual that the test did or did not estimate well (ibid., pp. 27–29, 39–44). The operational definition in Eq. (1) is typical of the individual, the specific test, and the administration conditions (ibid., pp. 39). From Eqs. (1), (2), and (3), it follows that, based on one test administration, in a group of subjects, the expected measurement *E* error is 0 [$\mathcal {E}( E) =0$] and measurement error *E* covaries 0 with the true score *T* on the same test [$\sigma ( {E,}{T})$], and with the true score on a different with test score *Y* [$\sigma ({E_{X},}{T_{Y}} )=0$] [ibid., p. 36, Theorem 2.7.1 (i), (ii), (iii), respectively]. In addition, assuming that the scores on two different tests with test scores *X* and *Y* are independently distributed for each person, it can be shown that across persons, the covariance between the measurement errors is 0; that is, $\sigma ({E_{X},}{E_{Y}})=0$ [ibid., Theorem 2.7.1 (iv), proof on p. 37]. We summarize these results by saying that measurement error covaries 0 with any other variable *Y*, not necessarily a test score, in which *E* is not included so that

4 $$\begin{aligned} \sigma \left( {E,}{Y}\right) =0. \end{aligned}$$

One may notice that for the same test, $\sigma \left( E,X \right) =\sigma _{E}^{2}\ge 0$, because *E* is part of *X*: $X=T+E$. Because measurements can be anything, in the context of a test consisting of *J* items, an item *j* ($j=1,\ldots ,J)$ also qualifies as a measure, with random variable $X_{j}$ representing the measurement value of the item, and $T_{j}$ and $E_{j}$ representing item true score and item random measurement error, respectively, so that $X_{j}=T_{j}+E_{j}$. Similarly, at the group level, $\mathcal {E}(E_{j})=0$, $\sigma ({E_{j},}{T_{j}})=0$, $\sigma ({E_{j},}{T_{k}})=0$, and $\sigma ({E_{j},}{E_{k}})=0$.

Let the test score be the sum of the item scores,

5 $$\begin{aligned} X=\sum \nolimits _{j=1}^J X_{j}. \end{aligned}$$

The reliability of a measurement value, denoted $\rho _{XX^{'}}$, is a group characteristic, which is defined as follows. Two tests with test scores *X* and $X^{'}$ are parallel when they have the next two properties: (1) $T_{i}=T_{i}^{'}$, for all individuals *i*, and (2) for variances, $\sigma _{X}^{2}=\sigma _{X^{'}}^{2}$, at the group level. From this definition, save for two cases, one can derive that parallel tests have exactly the same formal properties. This follows from the definition that measurement error is random. The exceptions are that at the level of the tested individual, in general, $E_{i}\ne E_{i}^{'}$, so that $X_{i}\ne X_{i}^{'}$, and that the distributions of *E* and $E^{'}$ can be different with the restrictions that their means are 0 [i.e., $\mathcal {E}\!\left( E \right) =\mathcal {E}\!\left( E^{'} \right) =0]$ and their variances equal (i.e., $\sigma _{E}^{2}=\sigma _{E^{'}}^{2})$; see Lord & Novick (1968, p. 46). Lord and Novick (1968, p. 47) define replications using the concept of linear experimental independence (ibid., p. 45), which says that the first measurement does not affect the first moment of the test scores from the second measurement, and hence, the two measurements are uncorrelated. Linearly experimentally independent measurements that have properties (1) and (2) of parallel measurements qualify as replications (ibid., p. 47).

The reliability definition is based on this idea of replicability—what would happen if I would repeat the measurement procedure under the same circumstances?—and is defined as the product-moment correlation between two parallel tests administered in a population of respondents. Reliability $\rho _{XX^{'}}$ can be shown to be equal to the proportion of the test-score variance, $\sigma _{X}^{2}$ (or, equivalently, $\sigma _{X^{'}}^{2})$, that is true-score variance, $\sigma _{T}^{2}$ (or, equivalently, $\sigma _{T^{'}}^{2})$, so that

6 $$\begin{aligned} \rho _{XX^{'}}=\frac{\sigma _{T}^{2}}{\sigma _{X}^{2}}=\frac{\sigma _{T^{'}}^{2}}{\sigma _{X^{'}}^{2}}. \end{aligned}$$

Noting that from Eqs. (2) and (4), one can derive that

7 $$\begin{aligned} \sigma _{X}^{2}=\sigma _{T}^{2}+\sigma _{E}^{2}+2\sigma _{TE}=\sigma _{T}^{2}+\sigma _{E}^{2}, \end{aligned}$$

reliability can also be written as

8 $$\begin{aligned} \rho _{XX^{'}}=1-\frac{\sigma _{E}^{2}}{\sigma _{X}^{2}}=1-\frac{\sigma _{E^{'}}^{2}}{\sigma _{X^{'}}^{2}}. \end{aligned}$$

In this article, we will use the definition of parallel measures at the item level. Let $\sigma _{j}^{2}$ be the item-score variance for item *j*.

#### Definition 1

Two items *j* and *k* with scores $X_{j}$ and $X_{k}$ are parallel if:

9 $$\begin{aligned}&(1)\,T_{ij}=T_{ik},\hbox { for all individuals } i;\hbox { and} \end{aligned}$$

10 $$\begin{aligned}&(2)\, \sigma _{j}^{2}=\sigma _{k}^{2},\hbox { at the group level}. \end{aligned}$$

Let $\sigma ({X_{j},}{X_{k}})=\sigma _{jk}$ denote the covariance. First, notice that, in general, because $\sigma ({E_{j},}{E_{k}})=0$, it follows that for groups, $\sigma ({T_{j},}{T_{k}})=\sigma _{jk}$. Using this result, property (1) in the definition of parallel items implies for three items *j*, *k*, and *l*, that $\sigma _{jk}=\sigma _{jl}=\sigma _{kl}$. Hence, parallel items have equal inter-item covariances. Combining this result with property (2) in the definition of parallel items implies that the inter-item correlations are also equal: $\rho _{jk}=\rho _{jl}=\rho _{kl}$.

The discussion so far suffices to present (without proof) the inequality

11 $$\begin{aligned} \alpha =\frac{J}{J-1}\times \frac{\sum \sum \nolimits _{j\ne k} \sigma _{jk} }{\sigma _{X}^{2}}\le \frac{\sigma _{T}^{2}}{\sigma _{X}^{2}}=\rho _{XX^{'}}, \end{aligned}$$

with

12 $$\begin{aligned} \sigma _{X}^{2}=\sum \nolimits _{j=1}^J \sigma _{j}^{2} +\sum \sum \nolimits _{j\ne k} \sigma _{jk}. \end{aligned}$$

Before we present this important result as a theorem, we define a weaker form of equivalence than parallelism, which is essential $\tau$-equivalence (Lord & Novick, 1968, p. 50, Definition 2.13.8; note: $\tau$ stands for the true score).

#### Definition 2

Two items with scores $X_{j}$ and $X_{k}$ are essentially $\tau$-equivalent if, for scalar $b_{jk}$,

13 $$\begin{aligned} T_{ij}=T_{ik}+b_{jk}. \end{aligned}$$

Definition 2 implies that, unlike parallel items, essential $\tau$-equivalent items do not necessarily have the same item-score variances, so that in general albeit not necessarily, $\sigma _{j}^{2}\ne \sigma _{k}^{2}$. Because true scores of essentially $\tau$-equivalent items differ only by an item-pair-dependent additive constant, and additive constants do not influence variances and covariances, for three essentially $\tau$-equivalent items *j*, *k*, and *l* we have that $\sigma _{T_{j}}^{2}=\sigma _{T_{k}}^{2}=\sigma _{T_{l}}^{2}$ and $\sigma _{jk}=\sigma _{jl}=\sigma _{kl}$. Combining equal inter-item covariances with item-score variances that can be unequal, essential $\tau$-equivalent items do not necessarily have equal inter-item correlations. Obviously, parallelism is a special case of essential $\tau$-equivalence when $b_{jk}=0$ and item-score variances are equal, $\sigma _{j}^{2}=\sigma _{k}^{2}$.

Next, we present the inequality relation of coefficient $\alpha$ and test-score reliability $\rho _{XX^{'}}$ as a theorem.

#### Theorem

Coefficient $\alpha$ is a lower bound to the reliability of the test score; that is,

14 $$\begin{aligned} \alpha \le \rho _{XX^{'}}, \end{aligned}$$

with equality $\alpha =\rho _{XX^{'}}$ if and only if items or test parts on which coefficient $\alpha$ is based are essentially $\tau$-equivalent.

#### Proof

See, for example, Novick and Lewis (1967) and Ten Berge and Sočan (2004), and Lord and Novick (1968, p. 90, Corollary 4.4.3b); also, see Guttman (1945).

Thus, based on essential $\tau$-equivalence, equal inter-item covariances are a necessary condition for equality $\alpha =\rho _{XX^{'}}$, meaning that varying covariances indicate that strict inequality $\alpha <\rho _{XX^{'}}$ holds. It has been suggested (e.g., Dunn et al., 2014) that greater variation of covariances produces a greater difference between $\alpha$ and $\rho _{XX^{'}}$, implying that $\alpha$ is less informative about $\rho _{XX^{'}}$. Greater variation of inter-item covariances may suggest a multi-factor structure of the data, meaning that one may consider splitting the item set into subsets that each assesses an attribute unique to that subset. Because each item subset is a separate test, all we say in this article applies to each item subset as well.

A third definition of item equivalence is that of congeneric items (e.g., Bollen, 1989; Jöreskog, 1971; Raykov, 1997a,1997b), often used in the FA context and defined as follows.

#### Definition 3

Two items with scores $X_{j}$ and $X_{k}$ are congeneric if, for scalars $a_{jk}$ and $b_{jk}$,

15 $$\begin{aligned} T_{ij}=a_{jk}T_{ik}+b_{jk}. \end{aligned}$$

Compared to congeneric items, essential $\tau$-equivalence is more restrictive with $a_{jk}=1$ for all $j\ne k$. The covariances of congeneric items *j*, *k*, and *l*, which are $a_{jk}\sigma _{jk}$, $a_{jl}\sigma _{jl}$, and $a_{kl}\sigma _{kl}$, are obviously different from one another when item-pair-dependent scalars $a_{jk}$, $a_{jl}$, and $a_{kl}$ are different. One may notice that inter-item correlations are also different. Hence, for congeneric items we have strictly $\alpha <\rho _{XX^{'}}$.

Finally, we rewrite coefficient $\alpha$ to a form that provides much insight in its relationship to the dimensionality of the data. Let ${{\bar{\sigma }}}$ be the mean of the $\frac{1}{2}J(J-1)$ inter-item covariances $\sigma _{jk}$, then

16 $$\begin{aligned} \alpha =\frac{J^{2}{{\bar{\sigma }}}}{\sigma _{X}^{2}}. \end{aligned}$$

We note that coefficient $\alpha$ depends on the mean inter-item covariance but not on the distribution of the inter-item covariances. This is important, because the distribution and not its mean holds the information about the dimensionality of the item set. For example, a set of inter-item covariances may have a mean equal to ${{\bar{\sigma }}}=c$, *c* is a number, and many different sets of varying inter-item covariances representing various factor structures may have this same mean ${{\bar{\sigma }}}=c$. As an extreme case, all inter-item covariances may be equal to $\sigma _{jk}=c$, which represents essential $\tau$-equivalence, and thus we have $\bar{\sigma }=\sigma _{jk}=c$. These observations make clear that a particular $\alpha$ value can represent numerous cases of multidimensionality with essential $\tau$-equivalence as a limiting case, thus showing that $\alpha$ is uninformative of data dimensionality.

### Discrepancy between Coefficient ${\alpha }$ and Reliability ${\rho }_{{{X}}{{X}}^{{'}}}$

*Discrepancy* refers to the difference between two parameters, such as $\alpha -\rho _{XX^{'}}$; if items are essentially $\tau$-equivalent, then discrepancy $\alpha -\rho _{XX^{'}}=0$, but given that essential $\tau$-equivalence fails for real tests, in practice, discrepancy $\alpha -\rho _{XX^{'}}<0$. We notice that test constructors often successfully aim for high reliability when the test is used to diagnose individuals, say, at least .8 or .9 (Oosterwijk, Van der Ark, & Sijtsma, 2019), rendering discrepancy small for many real tests. It is of interest to know when discrepancy is large negative so that coefficient $\alpha$ is rather uninformative of reliability and should be re-assessed or ignored. Discrepancy is especially large when individual items have little if anything in common (Miller, 1995) so that ${{\bar{\sigma }}}\approx 0$ [Eq. (16)], but their scores are highly repeatable across hypothetical replications, meaning $\sigma _{E}^{2}\approx 0$, so that $\rho _{XX^{'}}$ is close to 1 [Eq. (8)]. An artificial, didactically useful, and admittedly nonsensical example makes the point clear. We consider a sum score of measures of shoe size, intelligence, and blood sugar level. In a group of adults, we expect little association between the three measures, resulting in ${{\bar{\sigma }}}\approx 0$ and thus a low $\alpha$ value, perhaps $\alpha \approx 0$ [Eq. (16)]. However, across hypothetical replications, we expect little variation in the results per person, hence, we expect little random measurement error, $\sigma _{E}^{2}\approx 0$, and a high reliability, $\rho _{XX^{'}}\approx 1$ [Eq. (8)]. Thus, discrepancy of coefficient $\alpha$ and reliability $\rho _{XX^{'}}$ is large negative, almost $\alpha -\rho _{XX^{'}}=-1$, and the conclusion must be that coefficient $\alpha$ is uninformative of reliability $\rho _{XX^{'}}$. The usefulness of the example is that it shows that cases of extremely pronounced multidimensionality produce large discrepancy. The example also suggests that a real test that one constructed skillfully is not this extremely multidimensional. For less extreme and substantively more sensible cases of multidimensionality, we suggest one considers separate subtests that are homogeneous by content, each subtest showing small discrepancy $\alpha -\rho _{XX^{'}}$.

This is the right place to consider a popular FA perspective on reliability. This FA perspective argues that if one replaces the true-score variance in the CTT reliability definition [Eq. (6)] with the common-factor variance resulting in an FA reliability definition denoted $\rho _{XX}^{*}$, discrepancy is smaller when one compares coefficient $\alpha$ with $\rho _{XX}^{*}$ rather than $\rho _{XX^{'}}$ (Bentler, 2009). The broader context of the FA approach is that it enables the accommodation of multidimensionality and correlated errors in a reliability analysis. Thus, the approach should convince us to adopt the FA definition of reliability and reject the CTT reliability. However, we should realize that unless one assumes that factor-model variance equals true-score variance, the FA reliability definition is different from the CTT reliability definition [Eq. (6)] and the consequence of this inequality is that FA reliability does not equal the product-moment correlation of two parallel tests, $\rho _{XX^{'}}$. Thus, by adopting the FA definition of reliability the price one pays for smaller discrepancy is that reliability no longer is a measure for repeatability but a measure for proportion of test-score variance that is factor-model variance, for example, common-factor variance. This raises the question whether one is still dealing with reliability or with another quantity. Irrespective of this issue, we will show that in this case, the chosen factor model still is a CTT model. Next, we focus on discrepancy, $\alpha -\rho _{XX}^{*}$. Before we do, we should mention that Bentler (2009, p. 138) uses notation $\rho _{xx}$ for the FA definition and $\rho _{XX}^{*}$ for the CTT definition, which refers to our definition in Eq. (6). Because for the CTT definition the common notation is $\rho _{XX^{'}}$, we will stick to it and use $\rho _{XX}^{* }$ for the FA definition. We do not use a prime in the FA definition, because parallel tests no longer play a role in that context. Another word of caution refers to the fact that the next exercise is entirely theoretical; the model discussed is not estimable.

Bentler (2009) suggested splitting score $X_{j}$ for item *j* in the sum of a common factor, an item-specific factor, and a random error, so that true score $T_{j}$ is the sum of the common factor and an item-specific factor. Then, replacing the true score with the common factor in the relevant equations in the reliability definition [Eq. (8)], Bentler argued that coefficient $\alpha$ based on the common factor is a lower bound to the reliability based on the common factor, $\rho _{XX}^{*}$. He also showed that $\rho _{XX}^{*}$ is a lower bound to the reliability based on the true score [Eq.  (6)]; hence, $\alpha \le \rho _{XX}^{*}\le \rho _{XX^{'}}$. It follows that adopting Bentler’s model, discrepancy $\alpha -\rho _{XX}^{*}$ is smaller than it is in the CTT context, where one considers $\alpha -\rho _{XX^{'}}$. On the other hand, we show that although the terminology of item-specific factors suggests that one has to treat this score component separate of the common factor and the random error, the item-specific factor behaves mathematically as if it were random measurement error. The effect is that by introducing the item-specific factor, random measurement-error variance increases, and hence, true-score variance decreases. Thus, common-factor reliability equals true-score reliability, and the model does not change discrepancy; that is, $\alpha -\rho _{XX}^{*}=\alpha -\rho _{XX^{'}}$.

To see how this works, following a suggestion Bentler made, we define the common factor, such that $C_{j}=a_{j}\theta$, where $\theta$ is the item-independent factor and $a_{j}$ the item’s loading. Thus, the common factor $\theta$ depends on the specific items through the item loadings $a_{j}$. Bollen (1989, pp. 218-221) proposed the factor model $X_{j}=b_{j}+a_{j}\theta +\delta _{j}$, where $T_{j}=b_{j}+a_{j}\theta$ and $\delta _{j}$ is a residual including random measurement error, and derived a corresponding reliability coefficient. Mellenbergh (1998) assumed $\delta _{j}=E_{j}$ and studied the one-factor model $X_{j}=b_{j}+a_{j}\theta +E_{j}$. Moreover, he proposed a reliability coefficient for the estimated factor score ${{\hat{\theta }}}$ rather than test score *X*. We follow Bentler’s discussion and use his notation. Then, in addition to common factor $C_{j}$, the item-specific factor is denoted $S_{j}$, which is unique to one item, and the random measurement error is denoted $E_{j}$ [Eq. (2)]. In Bollen’s model, the item-specific component would be part of $\delta _{j}=S_{j}+E_{j}$, whereas in Mellenbergh’s model, it would be ignored, resulting in $\delta _{j}=E_{j}$. Bentler assumed that the three score components $C_{j}$, $S_{j}$, and $E_{j}$ do not correlate. For person *i*, the resulting model is a factor model, equal to

17 $$\begin{aligned} X_{ij}=C_{ij}+S_{ij}+E_{ij}. \end{aligned}$$

For a test score defined as the sum of the item scores [Eq. (5)], we also have $C=\sum \nolimits _{j=1}^J C_{j}$, $S=\sum \nolimits _{j=1}^J S_{j}$, and $E=\sum \nolimits _{j=1}^J E_{j}$. An alternative definition of reliability, in fact, an FA definition, then is

18 $$\begin{aligned} \rho _{XX}^{*}=\frac{\sigma _{C}^{2}}{\sigma _{X}^{2}}=1-\frac{\sigma _{S}^{2}+\sigma _{E}^{2}}{\sigma _{X}^{2}}. \end{aligned}$$

Because this reliability definition focuses on the common factor rather than the dimension-free true score *T*, Bentler considered $\rho _{XX}^{*}$ an appropriate coefficient of internal consistency, whereas he considered the classical coefficient $\rho _{XX^{'}}$ inappropriate for this purpose. Thus, in Bentler’s conception, internal consistency refers to unidimensionality operationalized by a common factor. He showed that in the factor model in Eq. (17), coefficient $\alpha$ is a lower bound to $\rho _{XX}^{*}$, and that $\rho _{XX}^{*}$ is a lower bound to the classical $\rho _{XX^{'}}$. Consequently, we have $\left| \alpha -\rho _{XX}^{*} \right| \le \left| \alpha -\rho _{XX^{'}} \right|$. The reason for larger discrepancy with respect to $\rho _{XX^{'}}$ is that the CTT approach ignores item-specific score components that are systematic across a group of people, so that $\mathcal {E}(S_{j})\ne 0$, but correlate 0 with other score components. The FA approach to reliability is of special interest to us, which is why we follow Bentler’s line of reasoning and notice the following.

Because both score components *S* and *E* are uncorrelated with each other and with common factor *C*, at the model level they show the same correlation behavior, and even though one can speak of a score component *S* that has an interpretation different from random measurement error, in Bentler’s approach *S* and *E* cannot be distinguished *mathematically*. We notice that the general result $\mathcal {E}(S_{j})\ne 0$ and $\mathcal {E}( E_{j})=0$ do not play a role in the derivations; hence, we can ignore possible conceptual differences between $S_{j}$ and $E_{j}$ and treat $S_{j}$ as a random error component. We combine *S* and *E* as residual $\epsilon =S+E$, with $\sigma _{\epsilon }^{2}=\sigma _{S}^{2}+\sigma _{E}^{2}+2\sigma _{SE}$, in which $\sigma _{SE}=0$ by definition, and it follows immediately that

19 $$\begin{aligned} \sigma _{\epsilon }^{2}\ge \sigma _{E}^{2}\Longrightarrow \rho _{XX}^{*}=1-\frac{\sigma _{\epsilon }^{2}}{\sigma _{X}^{2}}\le 1-\frac{\sigma _{E}^{2}}{\sigma _{X}^{2}}=\rho _{XX^{'}}. \end{aligned}$$

Because $\sigma _{\epsilon }^{2}=\sigma _{S}^{2}+\sigma _{E}^{2}$, from Eq. (19) and following Bentler (2009, Eq. 3) we conclude that

20 $$\begin{aligned} \rho _{XX}^{*}+\frac{\sigma _{S}^{2}}{\sigma _{X}^{2}}=\rho _{XX^{'}}, \end{aligned}$$

with equality

21 $$\begin{aligned} \rho _{XX}^{*}=\rho _{XX^{'}}\Longleftrightarrow \sigma _{S}^{2}=0. \end{aligned}$$

The result in Eq. (21) shows the conditions for which CTT reliability $\rho _{XX^{'}}$ [Eq. (6)] and Bentler’s factor-model reliability $\rho _{XX}^{*}$ [Eq. (18)] are equal. We will use this result after we have considered the condition for which $\alpha =\rho _{XX}^{*}$ and how this condition reduces to essential $\tau$-equivalence when $\rho _{XX}^{*}=\rho _{XX^{'}}$.

Rather than reiterating Bentler’s proof, which follows a different trajectory, we notice that mathematically, for the proof that $\alpha \le \rho _{XX}^{*}$ one does not distinguish the factor model [Eq. (17)] from the CTT model [Eq. (3)] in ways that are essential for the proof. The only difference is that residual-error variance, $\sigma _{\epsilon }^{2}$, is at least as great as random measurement error variance, $\sigma _{E}^{2}$ (i.e., $\sigma _{\epsilon }^{2}\ge \sigma _{E}^{2})$; hence, given fixed test-score variance, we find that $\alpha \le \rho _{XX}^{*}$ holds. It is paramount noticing that all that the use of the residual variance shows is that a greater error variance here defined as $\sigma _{\epsilon }^{2}$ but mathematically behaving like $\sigma _{E}^{2}$ in CTT, reduces reliability. Thus, it holds that

22 $$\begin{aligned} \alpha \le \rho _{XX}^{*}\le \rho _{XX^{'}}. \end{aligned}$$

We saw already that the second inequality becomes an equality if $\sigma _{S}^{2}=0$, and then, coefficient $\alpha$ again is a lower bound to reliability $\rho _{XX}^{*}=\rho _{XX^{'}}$, with equality if the items are essential $\tau$-equivalent. When does $\alpha =\rho _{XX}^{*}$?

To establish the condition for which $\alpha =\rho _{XX}^{*}$, we consider three items *j*, *k*, and *l* (also, see Bentler, 2009). Similar to essential $\tau$-equivalence, we define the concept of essential *C*-equivalence. By definition, the common factor components of the items must be essentially *C*-equivalent, common factor *C* replacing true score *T* (or $\tau )$; that is, for items *j* and *k*, we define $C_{ij}=C_{ik}+d_{jk}$, $d_{jk}$ is an item-pair-dependent scalar. Definitions are similar for item pairs *j* and *l*, and *k* and *l*. First, we notice that $\sigma ({C_{j},}{C_{k}})=\sigma (C_{k}+d_{jk},C_{k})=\sigma _{C_{k}}^{2}$, and replacing roles for items *j* and *k*, we find $\sigma _{C_{j}}^{2}=\sigma _{C_{k}}^{2}$, and extending results to all three items, we find $\sigma _{C_{j}}^{2}=\sigma _{C_{k}}^{2}=\sigma _{C_{l}}^{2}$. Second, because by assumption, different score components correlate 0 within and between items, and because scalars appearing in a sum do not affect covariances, writing $\sigma \!\left( C_{j},C_{k} \right) =\sigma (X_{j}-S_{j}-E_{j},X_{k}-S_{k}-E_{k}-d_{jk})=\sigma _{jk}$, and for the other item pairs we find $\sigma ({C_{j},}{C_{l}})=\sigma _{jl}$ and $\sigma ({C_{k},}{C_{l}})=\sigma _{kl}$. Combining results for the variances and the covariances, we find

23 $$\begin{aligned} \sigma _{jk}=\sigma _{jl}=\sigma _{kl}. \end{aligned}$$

Hence, essentially *C*-equivalent items have equal inter-item covariances. For items, the common factor model equals $X_{j}=C_{j}+\epsilon _{j}$, $X_{k}=C_{k}+\epsilon _{k}$, and $X_{l}=C_{l}+\epsilon _{l}$, and for essentially *C*-equivalent items, there are no restrictions on the variances of the residuals, so that, in general, $\sigma _{\epsilon _{j}}^{2}\ne \sigma _{\epsilon _{k}}^{2}\ne \sigma _{\epsilon _{l}}^{2}$, including equality signs as a possibility. Consequently, as with essentially $\tau$-equivalent items, inter-item correlations are not necessarily equal. Another way to look at essential *C*-equivalence is to use the model $C_{j}=a_{j}\theta$, and notice that

24 $$\begin{aligned} \sigma _{C_{j}}^{2}=\sigma _{C_{k}}^{2}\Longrightarrow a_{j}^{2}\sigma _{\theta }^{2}=a_{k}^{2}\sigma _{\theta }^{2}, \,\hbox {hence}, a_{j}=a_{k}. \end{aligned}$$

From this result, one can deduce that essentially *C*-equivalent items, as they are defined here in terms of a common factor model with item-specific factors, have equal loadings. Thus, the mathematical conditions for $\alpha =\rho _{XX}^{*}$ are identical to those for $\alpha =\rho _{XX^{'}}$, emphasizing that the CTT framework fully operates here.

Thus, we have shown that (1) item-specific factors behave like random measurement error in CTT, so that $\rho _{XX}^{*}=\rho _{XX^{'}}$, and (2) $\alpha =\rho _{XX}^{*}$ if and only if items are essentially *C*-equivalent, which is consistent with essential $\tau$-equivalence in CTT. Ignoring the different terminology, we conclude that reliability based on the common-factor model [Eq. (17)] simply is CTT reliability, common-factor variance $\sigma _{C}^{2}$ replacing true-score variance $\sigma _{T}^{2}$ and residual variance $\sigma _{\epsilon }^{2}$ including item-specific factor variances $\sigma _{S}^{2}$ replacing random measurement-error variance $\sigma _{E}^{2}$.

### Bias of Sample Estimate ${{\hat{\alpha }}}$

If one estimates coefficient $\alpha$ from a sample of size *N*, substituting parameter item-score variances $\sigma _{j}^{2}$ by sample $S_{j}^{2}$ and parameter inter-item covariances $\sigma _{jk}$ by sample $S_{jk}$ resulting in estimate ${{\hat{\alpha }}}$, then in some samples ${{\hat{\alpha }}}$ may be larger than true reliability (Verhelst, 1998). This is a common result of sampling error, but it is not a typical property of coefficient $\alpha$.

**Fig. 1:**
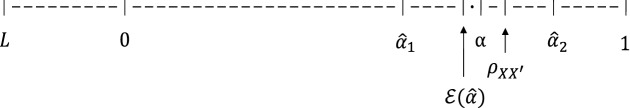
Scale for coefficient $$\alpha $$ ($$L\le \alpha \le 1$$; $$L<0)$$ and reliability $$\rho _{XX^{'}}$$ ($$0\le \rho _{XX^{'}}\le 1)$$. Estimates like $${{\hat{\alpha }}}_{2}$$ exceed reliability $$\rho _{XX^{'}}$$. Expectation $$\varepsilon ({{\hat{\alpha }}})$$ is smaller than parameter $$\alpha $$ (dot . suggests $$\varepsilon \left( {{\hat{\alpha }}} \right) -\alpha $$ is usually small).

If one considers the mean of sampling estimate ${{\hat{\alpha }}}$ across random samples of fixed size *N*, denoted $\mathcal {E}({{\hat{\alpha }}})$, then $\mathcal {E}(\hat{\alpha })-\alpha$ is the bias of ${{\hat{\alpha }}}$ relative to parameter $\alpha$. Figure 1 clarifies the bias for coefficient $\alpha$ and reliability $\rho _{XX^{'}}$. For essentially $\tau$-equivalent items and normally distributed true scores and measurement errors, using results presented by Feldt (1965), Verhelst (1998, p. 21) showed that estimate ${{\hat{\alpha }}}$ is negatively biased with respect to coefficient $\alpha$ by means of the expected value,

25 $$\begin{aligned} \mathcal {E}\!\left( \frac{1-{{\hat{\alpha }}}}{1-\alpha } \right) =\frac{N-1}{N-3}, \quad N>3. \end{aligned}$$

Hence, on average estimate ${{\hat{\alpha }}}$ underestimates parameter $\alpha$. As *N* grows,

26 $$\begin{aligned} \mathop {\lim }\limits _{N \rightarrow \infty }\left( \frac{N-1}{N-3} \right) =1, \end{aligned}$$

and already, for modest *N*, the bias soon is negligible.

Given less strict conditions than essential $\tau$-equivalence and using data generated based on various parameter choices for a data-simulation model, Oosterwijk (2016, p. 53) reported negative bias of ${{\hat{\alpha }}}$ relative to $\alpha$ for some models (i.e., $\overline{{\hat{\alpha }}} -\alpha <0$, $\overline{{\hat{\alpha }}}$ is the mean of ${{\hat{\alpha }}}$ acrosss samples). Moreover, he did not find positive bias for other models. For covariance matrices generated under a single-factor model, Pfadt et al. (2021) found that mean ${{\hat{\alpha }}}$ (i.e., $\overline{{\hat{\alpha }}} )$ showed negative bias that decreased to nearly 0 as sample size grew from $N=50$ to $N=500$.

We did additional analyses on data generated from a single-factor model for varying mean inter-item correlation, test length, and sample size, and 1,000 replicated data sets in each design cell. Table 1 shows maximum negative mean bias equal to $-0.00197$ and maximum positive mean bias equal to 0.00008. Assuming normality, negative mean bias was significant more often than expected based on the null hypothesis of no bias, thus supporting the theoretical negative bias result in Eq. (25) for finite sample size. Positive mean bias was never significant. These results provide us with confidence that estimate ${{\hat{\alpha }}}$ is negatively biased with respect to population $\alpha$, albeit only mildly.

The confirmation that estimate ${{\hat{\alpha }}}$ is not positively biased with respect to $\alpha$ is important, because, if large enough, a positively biased estimate ${{\hat{\alpha }}}$ could also systematically overestimate reliability $\rho _{XX^{'}}$, which is at least as large as coefficient $\alpha$. However, it does not. Because reliability $\rho _{XX^{'}}$ is of more interest to us than lower bound coefficient $\alpha$, we are primarily interested in the degree to which $\mathcal {E}({{\hat{\alpha }}})$ deviates from reliability $\rho _{XX^{'}}$. We define the difference $\mathcal {E}(\hat{\alpha })-\rho _{XX^{'}}$ as the bias of estimate ${{\hat{\alpha }}}$ with respect to reliability $\rho _{XX^{'}}$. Because we found absence of positive bias of estimate ${{\hat{\alpha }}}$ with respect to $\alpha$ (Table 1) and because $\alpha \le \rho _{XX^{'}}$, it seems safe to conclude that estimate ${{\hat{\alpha }}}$ is negatively biased with respect to reliability $\rho _{XX^{'}}$.

By the lower bound theorem, discrepancy $\alpha -\rho _{XX^{'}}$ is non-positive. The discrepancy depends on the distribution of the item scores and the test score, which depend in complex ways on the properties of the items. For concrete cases, we do not know the magnitude of the discrepancy, only that parameter $\alpha$ cannot be larger than parameter $\rho _{XX^{'}}$. Studies using artificial examples (e.g., Sijtsma, 2009) suggest discrepancy varies considerably and can be large when data are multidimensional. Thus, it makes sense to use coefficient $\alpha$ and other lower bounds only when the data are approximately unidimensional (Dunn et al., 2014).

**Table 1:** Bias of $\alpha$ Estimate (${{\hat{\alpha }}}$) and (Standard Error).

$\bar{\uprho }$	*J*	*N*				
		100	500	1000	2000	5000
.3	20	$-$1.97* (0.40)	$-$0.21 (0.16)	$-$0.07 (0.11)	0 (0.08)	0.08 (0.05)
	50	$-$0.59* (0.14)	$-$0.09 (0.06)	$-$0.02 (0.04)	$-$0.02 (0.03)	$-$0.04 (0.02)
.5	20	$-$0.96* (0.17)	$-$0.08 (0.08)	$-$0.13* (0.06)	0.02 (0.04)	$-$0.02 (0.02)
	50	$-$0.22* (0.06)	$-$0.03 (0.02)	$-$0.04* (0.02)	$-$0.02 (0.01)	$-$0.01 (0.01)
.7	20	$-$0.19* (0.05)	$-$0.03 (0.02)	$-$0.01 (0.02)	$-$0.02* (0.01)	0.01 (0.01)
	50	$-$0.12* (0.03)	$-$0.02* (0.01)	0 (0.01)	$-$0.01 (0.01)	$-$0.01* (0)
.8	20	$-$0.14* (0.04)	$-$0.04* (0.02)	0 (0.01)	$-$0.01 (0.01)	0.01 (0.01)
	50	$-$0.06* (0.02)	$-$0.01 (0.01)	$-$0.01* (0.01)	0 (0)	0 (0)

*Note.* Bias $(\overline{\hat{\alpha }}-\alpha )$ for four values of mean inter-item correlation (${{\overline{\rho }}} )$, two values of test length (*J*), and five values of sample size (*N*), 1000 replications per design cell. Entries have to be multiplied by .001; for example, $-1.97$ (0.40) stands for $-0.00197$ (0.0040). Significance is indicated by “*” and was tested by checking whether the normal theory confidence interval contained value zero

## Two Critical Claims about Coefficient ${\alpha }$

Now that we have discussed the state of knowledge with respect to coefficient $\alpha$, we are ready for discussing the two claims often made with respect to coefficient $\alpha$ and often used to discourage people from using coefficient $\alpha$ and sometimes other CTT lower bounds as well. The claims are: (1) Essential $\tau$-equivalence is unlikely to hold for real data collected with a set of items; hence, coefficient $\alpha$ has negative discrepancy with respect to reliability, and therefore, coefficient $\alpha$ is not useful. (2) When one incorporates correlated errors in the FA model, theoretically, coefficient $\alpha$ can be greater than test-score reliability, again triggering the conclusion that coefficient $\alpha$ should not be used.

### Claim (1): Essential ${\tau }$-Equivalence is Unrealistic; Hence, Lower Bounds Must Not be Used

*All Models Are Wrong; What’s the Consequence for Coefficient* $\mathbf {\alpha }$ *?* Several authors (e.g., Cho, 2016; Cho & Kim, 2015; Dunn et al., 2014; Graham, 2006; Teo & Fan, 2013) have claimed that coefficient $\alpha$ is useful only if what they call the *model of essential* $\tau$ *-equivalence* provides the correct description of the data. The reason for this claim is that equality $\alpha =\rho _{XX^{'}}$ holds if and only if items or other test parts on which coefficient $\alpha$ is based are essentially $\tau$ equivalent. Before we move on, as an aside we note that for binary scored items with different proportions of 1-scores, essential $\tau$-equivalence fails by definition, implying that $\alpha <\rho _{XX^{'}}$ and coefficient $\alpha$ is a strict lower bound. Returning to Claim (1) and assuming it refers to continuous item scores, authors making the claim often use FA definitions of reliability and essential $\tau$-equivalence, formalizing the latter condition with item difficulty $b_{j}$ and item-independent loading *a* on common factor $\theta$, as

27 $$\begin{aligned} X_{j}=b_{j}+a\theta +E_{j}, \end{aligned}$$

with $X_{j}$ continuous. We agree with many of the commentaries on coefficient $\alpha$ that essential $\tau$-equivalence and the corresponding FA model [Eq. (27)] pose restrictive conditions for a method to satisfy, but we question whether this implies one should limit the usefulness of coefficient $\alpha$ to this condition. Although often not explicated in the commentaries, by implication the conclusion to dismiss coefficient $\alpha$ implies dismissing all other classical reliability lower bounds (e.g., Bentler & Woodward, 1980; Guttman, 1945; Ten Berge & Zegers, 1978) when their equality to reliability depends on the condition of essential $\tau$-equivalence. This perspective ignores the frequent usefulness of lower bounds in practice, and we will explain why we are not prepared to throw the baby out with the bath water.

Before we explain why lower bounds can be useful, we consider Box (1976) for his interesting and much acclaimed clarification that models do not fit data but can be useful approximations. His famous quote “All models are wrong, but some are useful” (Box & Draper, 1987, p. 424) is more than an aphorism and states that by their very nature models are idealizations meant to pick up salient features of the phenomenon under study rather than capture all the details. Essential $\tau$-equivalence originally was not proposed as a model but was derived as the mathematical condition for which $\alpha =\rho _{XX^{'}}$, but we agree one might as well consider it a model for item equivalence. However, like all other models the model of essential $\tau$-equivalence, using Box’s words, can only be wrong, and for real data we can safely conclude that, strictly, $\alpha <\rho _{XX^{'}}$. Does this mean that one cannot use coefficient $\alpha$ anymore? A conclusion like this would imply that, following Box, because essential $\tau$-equivalence or its FA version [Eq. (27)] is wrong by definition, one could not use CTT nor FA reliability methods in practice, but we expect that very few colleagues would be prepared to draw this conclusion. Models are wrong but when they fit by approximation, results based on those models may still be useful. In the context of this article, this observation applies to both essential $\tau$-equivalence and its FA version [Eq. (27)], and to all factor models that substantiate reliability estimation based on one of these factor models. Here, the question we discuss is whether parameter $\alpha$ having negative discrepancy with respect to parameter $\rho _{XX^{'}}$ can be useful in practice.

*Practical Considerations for Using Lower Bounds*. Suppose one assesses consumer goods or services with respect to quality criteria. One may think of treatment success rates of hospitals and the percentage of students attending a particular high school that are admitted by good to excellent universities, but also mundane indexes such as a car’s fuel consumption and a computer’s memory and speed. Consumers have a natural inclination to require high treatment success and admittance rates, low fuel consumption, and large memory and high speed. Similarly, researchers and test practitioners require highly reliable test scores, thus welcoming high sample values. Two practical situations in which a person may be inclined to hope for high reliability values occur when external parties require high reliability as one of the necessary conditions for providing a particular “reward.” One may think of a publisher requiring high reliability as one of the conditions for publishing a test and a health insurance company requiring similar conditions for reimbursing the costs of diagnosing a psychological condition.

In situations in which people have an interest in reporting high reliability values, one may argue that some restraint may be in order. Given the need for restraint, one may argue that coefficient $\alpha$ and other reliability methods having small negative discrepancy and small negative bias with respect to reliability $\rho _{XX^{'}}$ may even provide some protection against too much optimism. Greater discrepancy and bias provide more protection, but also provide little information about true reliability. For coefficient $\alpha$, discrepancy and bias tend to be small for tests containing items consistent with one attribute and having approximately the same psychometric quality. To avoid confusion, we do not argue with the common statistical preference for zero discrepancy and bias (e.g., Casella & Berger, 1990), but wish to emphasize that the availability of small-discrepancy reliability lower bounds helps to mitigate too much optimism about reliability, especially when the optimism is based on small samples.

Reporting reliability values that are too high due to small sample size can be avoided by using larger samples and for several methods $N\ge 500$ may be just enough, as we discuss next. Commentaries on coefficient $\alpha$ do not so much promote essential $\tau$-equivalence as a desideratum but rather expose essential $\tau$-equivalence as a model the items must satisfy for coefficient $\alpha$ to equal reliability $\rho _{XX^{'}}$ and to be useful. We argue next that for approximate unidimensionality, lower bounds such as coefficient $\alpha$ come rather close to reliability $\rho _{XX^{'}}$ and in samples that are large enough do not tend to overestimate $\rho _{XX^{'}}$, which we consider a virtue for a quality measure. These are strong arguments favoring these lower-bound coefficients for reliability estimation.

*Selection of Lower Bounds.* In addition to coefficient $\alpha$, several other lower bounds exist (Sijtsma & Van der Ark, 2021, provide an overview). Guttman (1945) presented six lower bounds, denoted coefficients $\lambda _{1}$ through $\lambda _{6}$, with $\lambda _{3}=\alpha$. Mathematically, $\lambda _{1}<\lambda _{3}(=\alpha )\le \lambda _{2}$, and $\lambda _{3}\left( =\alpha \right) <\lambda _{4}$, which is the maximum value of coefficient $\alpha$ for all possible splits of the test in two test halves. Ten Berge and Zegers (1978) proposed an infinite series of lower bounds, denoted $\mu _{m}$, $m=0, 1,\ldots$, so that $\mu _{0}\le \mu _{1}\le \mu _{2}\le \ldots$, and $\mu _{0}=\lambda _{3}=\alpha$ and $\mu _{1}=\lambda _{2}$. Woodward and Bentler (1978; Bentler & Woodward, 1980) proposed the greatest lower bound (GLB). All other lower bounds are smaller than the GLB. Next, for population results, we discuss lower bounds that have a large negative discrepancy with respect to reliability $\rho _{XX^{'}}$. For sample results, we consider lower bound *estimates* that are too large, because they show positive bias relative to parameter $\rho _{XX^{'}}$.

First, if a lower bound has a *large* negative discrepancy relative to reliability $\rho _{XX^{'}}$, it may be practically useless, simply because it provides little information about reliability other than that reliability is much greater. We already noticed that when data are highly multidimensional, coefficient $\alpha$ has large negative discrepancy and may not be useful. The opposite is not true; that is, values of coefficient $\alpha$ are uninformative of the dimensionality of the data. In fact, a low $\alpha$ may represent unidimensional data and a high $\alpha$ may represent multidimensional data; all is possible. Nevertheless, Miller (1995) argued that when low, $\alpha$ values might warn against different items representing partly different attributes. It may but then again, it may not, see the discussion on coefficient $\alpha$’s dependence on mean inter-item covariance related to Eq. (16). Miller (1995) was not wrong that low $\alpha$ may indicate multidimensionality, but our point is that it can also indicate anything else and based on $\alpha$ alone one cannot draw conclusions about the dimensionality of the data. We recommend researchers to use FA or item response theory (IRT) for identifying item subsets, and to use coefficient $\alpha$ to estimate reliability for each item subset.

Second, lower bounds based on algorithms optimizing certain method features may capitalize on chance and produce positively biased estimates even if their discrepancy is negative (which it is by definition). Such methods may not be useful in practice. Oosterwijk, Van der Ark, and Sijtsma (2017) found that theoretical lower bounds coefficient $\lambda _{4}$ and the GLB tend to capitalize on chance when estimated for generated data, both unidimensional and 2-dimensional, and tend to overestimate reliability $\rho _{XX^{'}}$. Between $78\%$ and $100\%$ of sample values were larger than $\rho _{XX^{'}}$ irrespective of sample size $50\le N\le 1000$, and especially for test length of 10 and 15 items. (Larger test lengths were not included.) Dimensionality had little impact on results, and proportions of overestimates were invariably high. These results demonstrate that one should use reliability methods such as coefficient $\lambda _{4}$ and GLB with great restraint. (Sijtsma, 2009, was still rather positive about the GLB, but later results suggested the GLB’s deficiencies.)

Oosterwijk et al. (2017) also found in simulated data that for unidimensionality, coefficient $\lambda _{2}$’s discrepancy did not exceed $-.002$, but for 2-dimensionality discrepancy could become as great as $-.072$. For unidimensionality, less than $50\%$ of the estimates ${{\hat{\lambda }}}_{2}$ exceeded reliability $\rho _{XX^{'}}$, and this percentage decreased as *N* increased. Because coefficient $\alpha$ is mathematically similar and only a little smaller than coefficient $\lambda _{2}$, based on experience often no more than .01, results for coefficient $\alpha$ may be similar to results for coefficient $\lambda _{2}$. Both coefficients $\alpha$ and $\lambda _{2}$ have the virtue of simplicity and produce quite good results, but more definitive results may be in order.

### Claim (2): Correlated Errors Cause Failure of the Lower Bound Theorem

*Conceptual Differences Between CTT and FA Approaches*. One cannot measure a psychological attribute without at the same time also recording skills, auxiliary attributes, and environmental constancies that affect people differentially. In other words, non-target attributes always contaminate psychological measurement implying that a measurement value is never a reflection of only the target attribute. Whereas CTT is blind to this reality and seeks to answer the question to what degree a set of measurement values, no matter their origins, is repeatable under the same circumstances, the FA approach to reliability seeks to disentangle target from non-target influences on measurement and define reliability based only on the target attribute. There are also FA approaches that are based on sets of target and non-target attributes. We already discussed Bentler’s approach (Bentler, 2009) that explicitly defined a common factor representing the target attribute, and non-target influences separated into item-specific systematic influences and random measurement error, all score components correlating zero.

The systematic non-target influences are sometimes called *systematic errors*, where the terminology of error suggests one rather wished the influences did not happen. Non-target influences need not correlate zero among one another and with target abilities. For example, visual-motor coordination and speed may play an auxiliary role when responding to typical maze items in an intelligence test that predominantly measures perceptual planning ability (Groth-Marnat, 2003, pp. 177-178). Children showing the same level on the target attribute of perceptual planning ability may obtain systematically different test scores when they show different levels of the non-target skills of visual-motor coordination and speed. When this happens, non-target influences affect inter-item covariances. CTT includes all systematic influences, both target and non-target, on item and test performance in the true score. In the example, the true score reflects not only perceptual planning ability but also visual-motor coordination and speed, and perhaps other influences as well. The vital difference between the FA and CTT approaches is that FA does not and CTT does ignore the test score’s composition. The FA perspective commits to identifying the factor structure of the item set and incorporate this structure in the reliability approach.

We consider the CTT and FA approaches to reliability as representing different perspectives on reliability. Whether one accepts including all systematic performance influences in the true score and defines reliability as the proportion of test-score variance that is true-score variance or separates target and systematic non-target influences and defines reliability as the proportion of common-factor variance (Bentler, 2009) or a variation thereof, is a matter of preference. The CTT perspective, perhaps not even as a conscious strategy, is that the measurement of, for example, perceptual planning ability can only exist in real life together with the simultaneous measurement of visual-motor coordination and speed. The FA perspective would thus isolate the common factor representing perceptual planning ability—a hypothesis one needs to investigate by means of additional validity research—and then estimate the proportion of test-score variance that is common-factor variance.

We think both stances are legitimate—taking the test performance for granted as it appears in real psychological measurement or separating the various influences to obtain a purer measure—but we also notice the following. First, when responding to items, people simply use auxiliary skills and attributes, react in particular ways to stimulus cues, and are distracted by many external cues, and are incapable of suppressing doing all of this when providing a response. Second, by replacing the true-score perspective with the common-factor perspective, one loses the interpretation of reliability as the correlation between two parallel tests representing replications. The FA approach to reliability does not answer the question what would happen when a group of people repeatedly takes the same test under the same circumstances.

*The Lower Bound Theorem Assumes Uncorrelated Errors*. Several authors discussed correlated errors (e.g., Cho & Kim, 2015; Dunn et al., 2014; Green & Hershberger, 2000; Green & Yang, 2009; Lucke, 2005; Rae, 2006; Teo & Fan, 2013). For example, Raykov (2001) assumed that non-target influences on the performance on several items cause correlated errors. An example is social desirability affecting the responses to some items in a personality inventory. Another example is the presence of noise in the testing facility as a characteristic of the test administration procedure. One could argue that such non-target influences necessitate a model that allows for correlated errors. An attempted proof, such as in Raykov (2001), that allows correlated errors does not arrive at the lower bound theorem, which is based on the assumption that errors do not correlate. Models assuming correlating errors lead to different reliability approaches.

CTT only distinguishes the true score and random measurement error, but in the preceding section, we argued that several attributes affect item performance, one usually targeted or intended and the others non-targeted or unintended and both assumed distinct from random measurement error. The essence of discussions about coefficient $\alpha$ allegedly not being a reliability lower bound is that authors are of the opinion that non-targeted attribute influences cannot be part of the true score and have a distinct position in a model, often as a systematic error component. A model implying correlated errors is the basis for studying whether coefficient $\alpha$ still is a lower bound under this alternative model (see, e.g., Raykov, 2001). It is not, because a model assuming only uncorrelated errors underlies the lower bound theorem.

Whereas this conclusion seems to let coefficient $\alpha$ off the hook, we acknowledge that researchers might come across a test situation that they suspect includes correlated errors and wonder whether to compute coefficient $\alpha$ or not. We argue that it is always admissible to compute coefficient $\alpha$ since we identified another misconception at play that seems to disqualify any reliability coefficient that cannot account for correlating errors. This misconception, in particular, is the assumption that each particular test allegedly has only one reliability. From this uniqueness assumption, it follows that if one administers the test to a group susceptible to social desirability or if one administers it in a noisy testing facility, the collected data are confounded and cannot produce the “correct” reliability. Hence, the need for correlated errors that allow the focus on the target influence and accommodate non-target influences to be included in the error term of the model. Then, focusing on the target influence would produce the correct reliability. However, this approach misses an important point. This point is that CTT reliability is defined for any combination of test, group to which it is administered, and administration procedure, and in each situation defined by test, group, and procedure, reliability has a unique value. Thus, reliability values are dependent on the triplet test, group, and procedure. From the perspective of CTT, there are no bad tests, groups responding unfortunately, and disrupted administration procedures; none plays a role in the model. All reliability does is express the degree to which test scores are repeatable, and it does this for all triplets of test, population, and procedure. Each triplet produces data resulting in different numerical values for coefficient $\alpha$ and reliability $\rho _{XX^{'}}$, and the lower bound theorem is always true at the population level. Driving this to the limit, if we consider the same group taking the same test in one condition with loud, disturbing background noise halfway through the test affecting performance on some items and in another condition without the noise, the conditions produce different reliabilities according to CTT.

Of course, we do not advocate using bad tests, blindly accepting non-target influences, and tainted administration procedures, but the fact remains that the lower bound theorem is true no matter the triplet of test, group, and procedure. Neither do we imply that one should not use test theories modeling the true score implying correlated errors; if one wishes, one should. CTT deals with true-score variance, $\sigma _{T}^{2}$, but does not decompose it. FA approaches to reliability decompose true-score variance and use the decomposition to derive interesting results for that model. Whereas CTT defines reliability as the correlation between two parallel tests, hence the degree to which a test score *X* is repeatable, FA defines reliability as the proportion of variance of test score *X* that the factor model one uses explains. McDonald (1999) proposed coefficient $\omega$ to estimate this reliability. Coefficient $\omega$ knows different versions corresponding to different factor models. We notice that there is great potential in the FA approach to reliability. For instance, Mellenbergh (1998) suggested FA reliability focusing on the estimated common factor score, ${{\hat{\theta }}}$, rather than the test score *X* as coefficient $\omega$ does. Focusing on the estimated factor score seems to be consistent with the FA approach in which the factor score seems to define the scale of interest.

We end with recommendations for researchers. First, if you simply wish to know the degree to which test scores obtained in a group following a particular administration procedure are repeatable, you may use a lower bound to CTT reliability, such as coefficient $\alpha$. Key to understanding this recommendation is that CTT reliability depends on any test administered to any group following any procedure, and that coefficient $\alpha$ computed from data collected in a specific situation is always a lower bound to reliability specific of that same situation. Second, if you have doubts about the quality of the test, its constituting items, or the administration procedure, you may choose to improve the test, the items, the administration procedure, or a combination and then estimate CTT reliability for the improved situation using a lower bound method. Third, if you wish to correct test performance by modeling target influences and non-target influences that you consider undesirable and then determine reliability free of the non-target influences, you may use coefficient $\omega$ for the factor model that fits the collected data.

## Discussion and Conclusions

In psychology and many other research areas, coefficient $\alpha$ is one of the most reported measures for test quality. In addition to having become one of the landmarks in scientific reference, coefficient $\alpha$ also has attracted much criticism. Despite the criticisms, researchers continue using coefficient $\alpha$, which we claim has value in estimating test-score reliability next to other methods.

We summarize the usefulness of coefficient $\alpha$ as: Coefficient $\alpha$ is a mathematical lower bound to the reliability of a test score; that is, $\alpha \le \rho _{XX^{'}}$ [Eq. (14)]. A few remarks are in order. The remarks pertain to population results and parameters, unless indicated otherwise.

- The lower bound theorem, $\alpha \le \rho _{XX^{'}}$, is a correct mathematical result from CTT.
- In samples, estimates of coefficient $\alpha$ follow a sampling distribution, and some estimates may be greater than reliability $\rho _{XX^{'}}$.
- In case of approximate unidimensionality (one factor), coefficient $\alpha$ is close to reliability, $\rho _{XX^{'}}$.
- In case of multidimensionality (multiple factors), coefficient $\alpha$ may be much smaller than reliability, $\rho _{XX^{'}}$.
- Coefficient $\alpha$ is not an index for internal consistency. In samples, we recommend using FA or IRT for identifying subsets of items and estimating coefficient $\alpha$ for each subset. This is really all there is to say about coefficient $\alpha$. We add the following recommendation:
- If one models reliability in an FA context, we recommend estimating the FA-tailored reliability coefficient $\omega$ or to estimate the reliability of the estimated factor score.

It is remarkable that colleagues have articulated and continue to articulate so many criticisms on coefficient $\alpha$. In this contribution, we have argued that a lower bound measure such as coefficient $\alpha$ but also coefficient $\lambda _{2}$ can be considered as a mild insurance policy against too much optimism about reliability. We have also argued that a lower bound theorem that was derived under certain conditions simply is true, and only when one changes the conditions will the theorem fail. A caveat to using CTT-based lower bounds is that in research, they may produce inflation of attenuation correction (Lord & Novick, 1968, p. 69). We are unaware of similar results for FA reliability.

We emphasize that there is nothing wrong with the FA approach, but also remind the reader that it is different from CTT. Briefly, in CTT, any score component that correlates with another score component contributes to the true-score variance, and all other score components that correlate zero with the item’s true score and other items’ error scores contribute to the error-score variance. CTT is uncritical about further subdivisions. However, the FA approach is critical by distinguishing a common factor from group factors and optional item-specific factors, thus splitting the true score variance into different parts and possibly assigning item-specific factors to the model’s residual. Different versions of coefficient $\omega$ reflect different factor models. Whether one uses CTT or FA is a matter of taste; both are mathematically consistent. Mixing up models may lead to false claims about the less preferred model and its methods, obviously something to avoid. The CTT definition of reliability, which expresses the degree to which two parallel tests or test replications correlate linearly [Eq.  (6)], is a valuable contribution to measurement, and coefficient $\alpha$ provides a lower bound that is useful when the test measures one dimension or factor by approximation.
